# Correlating Epidemiologic Trends with the Genotypes Causing Meningococcal Disease, Maryland

**DOI:** 10.3201/eid1003.020611

**Published:** 2004-03

**Authors:** M. Catherine McEllistrem, John A. Kolano, Margaret A. Pass, Dominique A. Caugant, Aaron B. Mendelsohn, Antonio Guilherme Fonseca Pacheco, Jafar Razeq, Lee H. Harrison, the Maryland Emerging Infections Program

**Affiliations:** * University of Pittsburgh Graduate School of Public Health and School of Medicine, Pittsburgh, Pennsylvania, USA; † Johns Hopkins University Bloomberg School of Hygiene and Public Health, Baltimore, Maryland, USA; ‡ World Health Organization Collaborating Centre for Reference and Research on Meningococci, Norwegian Institute of Public Health, Oslo, Norway; § University of Pittsburgh Graduate School of Public Health, Pittsburgh, Pennsylvania, USA; ¶ Maryland Department of Health and Mental Hygiene, Baltimore, Maryland, USA

**Keywords:** *Neisseria meningitidis*, molecular epidemiology, pulsed-field gel electrophoresis, multi-locus sequence typing, serogroup C, serogroup Y, bacteria, Centers for Disease Control and Prevention

## Abstract

Epidemic meningococcal infection is generally caused by single clones; whether nonepidemic increases in infection are clonal is unknown. We studied the molecular epidemiology of meningococcal infection during a period that the incidence increased in two age groups. Serogroup C and Y meningococcal isolates were analyzed by pulsed-field gel electrophoresis and multilocus sequence typing. From 1992 to 1999, 96.4% (27/28) of serogroup C isolates from persons 15–24 years of age were in clonal group 1, compared with 65.6% (21/32) of isolates from persons ≤14 years, and 64.3% (9/14) of isolates from adults ≥25 years (p ≤ 0.01). The proportion of clonal group 2 serogroup Y strains increased from 7.7% (1/13) in 1992 to 1993 to 52.0% (13/25) in 1998 to 1999 (p < 0.01). The nonepidemic age-specific increases in serogroup C meningococcal infection in Maryland were clonal in nature and the changes in serogroup Y incidence were associated with a shift in the genotypes of strains causing invasive disease.

Two important changes occurred in the epidemiology of meningococcal infection in Maryland and other parts of the United States during the 1990s. First, there was a substantial increase and subsequent decline in the incidence of meningococcal infections in persons ages 15 to 24 years in the absence of an epidemic in other age groups; most of the increase was caused by serogroup C infections (*1*,*2*). From 1990 to 1997, the incidence increased from 0.9 to 2.1 cases per 100,000 in this age group (p = 0.01) before declining to baseline in the late 1990s. Epidemic meningococcal disease is associated with an increasing incidence in adolescents and young adults and is usually caused by a single clone (*3*,*4*). Second, the incidence of meningococcal infection also increased steadily in adults >25 years of age during the 1990s (p = 0.03) (*1*).

The molecular epidemiology of *Neisseria meningitidis* infection has historically been characterized by using multilocus enzyme electrophoresis (MEE) (*5*,*6*). Due to the complicated nomenclature and labor-intensive nature of electrophoretic type (ET) determination, alternative methods have been pursued. Recently, multilocus sequence typing (MLST) of housekeeping genes has been shown to highly correlate with ET (*7*). Pulsed-field gel electrophoresis (PFGE) has also been shown to be a useful method for discriminating between sporadic and outbreak strains (*8*). We sought to determine whether the genotypes of *N. meningitidis* causing invasive disease correlated with the epidemiologic trends that were observed in Maryland during the 1990s.

## Methods

Active, laboratory- and population-based surveillance for invasive meningococcal infection from January 1, 1992, to December 31,1999, performed as part of the Maryland Bacterial Invasive Disease Surveillance project (BIDS), was the subject of this analysis (*1*,*9*). BIDS is the Active Bacterial Core Surveillance (ABCs) component of the multistate Emerging Infections Program Network coordinated by the Centers for Disease Control and Prevention (CDC) (*10*). The surveillance case definition was the isolation of *N. meningitidis* from a normally sterile body fluid from a Maryland resident (*1*).

Meningococcal serogroups were determined by the Maryland Department of Health and Mental Hygiene Laboratory Administrations and CDC. Serogrouping was repeated on isolates at the Norwegian Institute of Public Health when the PFGE pattern of a particular isolate was genetically more related to isolates of a different serogroup. If discrepant serogroup results were obtained, the serogroup that was most consistent with the isolate’s PFGE pattern was used in the analysis.

### PFGE

PFGE was performed as previously described (*11*). Briefly, equal amounts of bacterial suspension and 2% low-melting agarose (Sea Plaque, FMC Bioproducts, Rockland, ME) were pipetted into plug molds and incubated in ESP buffer (0.5M EDTA, 1% N-lauroyl sarcosine, 1 mg/mL proteinase K; pH 8.5–9.3) overnight at 50°C. After being washed 3 times with TE (Tris 0.5M EDTA) buffer at 37°C, the plugs were restricted with 20 U of *Nhe*I (New England Biolabs, Beverly, MA), 330 μg/mL of bovine serum albumin, and 200 μL of New England buffer #2 at 37°C overnight. PFGE was performed in a 1% agarose gel with the following run parameters: 1–30 s for 18 h, 5–9 s for 8 h at 14°C. After the gel was stained with ethidium bromide, the image was digitized on the Bio-Rad Gel Doc 2000 System (Bio-Rad, Hercules, CA). Dendrograms of genetic relatedness were created with Molecular Analyst/Multi-Analyst programs (Bio-Rad) by using the unweighted pair group method with arithmetic averages, and a position tolerance of 1.5%.

The cophenetic correlation, a measure of the correlation between the genetic relatedness represented on the dendrograms and the actual Dice coefficient–derived degree of relatedness, was calculated for the serogroup C and Y dendrograms. A meningococcal clone was defined as isolates with an indistinguishable PFGE pattern. A PFGE-based clonal group was defined as isolates with a ≤3-band difference (*12*) and/or ≥80% genetic relatedness on dendrogram (*8*).

### MLST

A subset of serogroup C and Y strains, selected to represent the range of PFGE types identified among our isolates, underwent MLST analysis. MLST was performed by using the following seven housekeeping genes (protein products are shown in parentheses): *abcZ* (putative ATP-binding cassette transporter), *adk* (adenylate kinase), *aroE* (shikimate dehydrogenase), *fumC* (fumarate hydratase), *gdh* (glucose 6-phosphate dehydrogenase), *pdhC* (pyruvate dehydrogenase subunit), and *pgm* (phosphoglucomutase) as previously described (*7*). The 470-bp fragments were amplified by polymerase chain reaction (PCR) and sequenced using an ABI Prism 377 (PE Applied Biosystems (Foster City, CA) with 5% Long Ranger Gels (FMC Bioproducts, Philadelphia, PA). DNA sequences were determined on both strands. Sequences were assembled with the Auto Assembler DNA Sequence Software Version 2.0 and consensus sequences compared with Sequence Navigator DNA and Protein Sequence Comparison Software (PE Applied Biosystems). Isolates with >5 alleles identical to sequence type (ST)-11 or ST-23 were defined as belonging to ST-11 or ST-23 complex, respectively (available from: http://neisseria.org/nm/typing/mlst/).

Statistical analyses and matrix manipulations were performed using SAS (Version 6.12; SAS Institute; Cary, NC) and R for Windows version 1.5.1 (available from: http://cran.r-project.org). Exact tests were performed using StatXact (Version 4.0.1, Cytel Software Corporation; Cambridge, MA). The Dice coefficients were the basis of the pairwise similarity analysis of the serogroup C isolates. In this analysis, the mean and median Dice coefficients were calculated separately for each age group and time period and the distributions of the Dice coefficients among the groups were compared using the Kruskal-Wallis rank sum test.

## Results

During the study period, 295 cases of meningococcal infection were reported (*1*), of which 258 (87.5%) were available for serogroup determination. Among these 258 isolates, 98 (38.0%) were serogroup Y, 83 (32.2%) were serogroup C, 51 (19.8%) were serogroup B, 12 (4.7%) were serogroup W-135, 2 (0.8%) were serogroup Z, and 1 (0.4%) was serogroup X; 11 (4.3%) could not be identified by serogroup.

**Figure 1:**
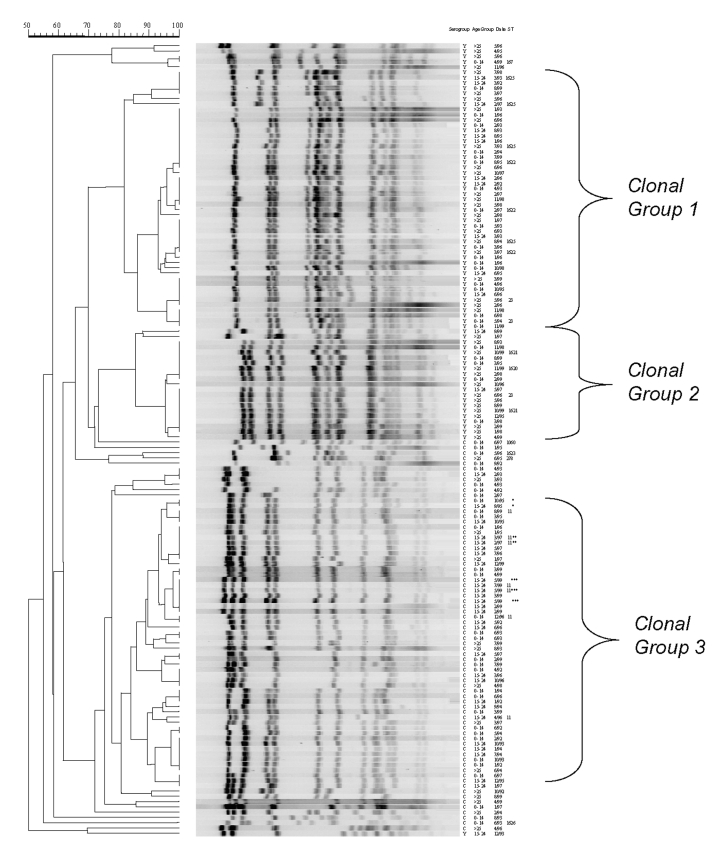
Pulsed-field gel electrophoresis patterns of meningococcal serogroup C strains isolated from persons <15 years of age (panel A), persons 15–24 years (panel B), and adults >25 years of age (panel C) during 1992–1999. Culture date and sequence type are listed to the right of the dendrogram.

Seventy-seven percent (57/74) of the serogroup C isolates were classified into clonal group 1 by PFGE. The cophenetic correlation for the serogroup C dendrogram was 89.2. There were no other predominant serogroup C clonal groups, and therefore, PFGE patterns not belonging to this clonal group were referred to as nonclonal group 1 strains. Over the 8-year period, 96.4% (27/28) of persons 15–24 years of age were infected with a serogroup C clonal group 1 strain compared with 65.6% (21/32) of persons aged >14 years (p <0.01), and 64.3% (9/14) of adults aged >25 years (p = 0.01). While the incidence was rising from 1992 to 1997 among persons aged 15–24 years, 95% (19/20) of persons in this age group were infected with a clonal group 1 strain compared with 57.7% (15/26) of persons <14 years (p <0.01) and 60.0% (6/10) of adults ∃>25 years (p = 0.03). From 1998 to 1999, the period during which the incidence in those aged 15 to 24 years had returned to baseline, 87.5% (7/8) of the serogroup C isolates from this age group were due to a single clone of clonal group 1 (Figure 1). This clone was only detected in 1999 among persons aged 15–24 years, including an outbreak of three cases (*13*). Another outbreak, which occurred in 1997 and consisted of two cases, was caused by a different serogroup C clonal group 1 clone (Figure 1). All clonal group 1 serogroup C strains, including those that caused both outbreaks, belonged to ST-11 (Tables1, 2). Among serogroup C isolates that underwent MLST, the nonclonal group 1 strains were unrelated to ST-11. Among adults >25 years, only 14 serogroup C isolates were available, and therefore, a meaningful trend analysis was not possible.

**Table 1:** PFGE and MLST results for selected serogroup C strains^a^

Age group (y)	Culture date	ST	No. alleles related to ST-11	ST-11 complex	PFGE interpretation	
15–24	3/97	11	7/7	Yes	Clonal group 1; 1997 outbreak	
15–24	2/97	11	7/7	Yes	Clonal group 1; 1997 outbreak	
15–24	7/99	11	7/7	Yes	Clonal group 1; 1999 clone	
15–24	5/99	11	7/7	Yes	Clonal group 1; 1999 outbreak	
<15	8/99	11	7/7	Yes	Clonal group 1	
<15	12/96	11	7/7	Yes	Clonal Group 1	
15–24	4/96	11	7/7	Yes	Clonal group 1	
<15	6/93	1,626	1/7	No	Nonclonal group 1	
<15	5/96	1,623	0/7	No	Nonclonal group 1	
<15	6/97	1,060	0/7	No	Nonclonal group 1	
>25	6/95	278	0/7	No	Nonclonal group 1	

^a^PFGE, pulsed-field gel electrophoresis; ST, sequence typing; MLST, multilocus sequence typing.

**Table 2:** PFGE and MLST results for selected serogroup Y strains

Age group	Culture date	ST	No. alleles related to ST-23	ST-23 complex	PFGE interpretation	
15–24	3/93	1,625	6/7	Yes	Clonal group 1	
15–24	2/97	1,625	6/7	Yes	Clonal group 1	
>25	8/94	1,625	6/7	Yes	Clonal group 1	
≥25	7/93	1,625	6/7	Yes	Clonal group 1	
<15	8/95	1,622	6/7	Yes	Clonal group 1	
<15	2/97	1,622	6/7	Yes	Clonal group 1	
>25	3/97	1,622	6/7	Yes	Clonal group 1	
<15	5/94	23	7/7	Yes	Clonal group 1	
>25	5/96	23	7/7	Yes	Clonal group 1	
> 25	6/96	23	7/7	Yes	Clonal group 2	
>25	11/99	1,620	5/7	Yes	Clonal group 2	
>25	10/99	1,621	6/7	Yes	Clonal group 2	
>25	10/99	1,621	6/7	Yes	Clonal group 2	
<15	4/99	167	0/7	No	Neither clonal group 1or 2	

^a^PFGE, pulsed-field gel electrophoresis; ST, sequence typing; MLST, multilocus sequence typing.

**Figure 2:**
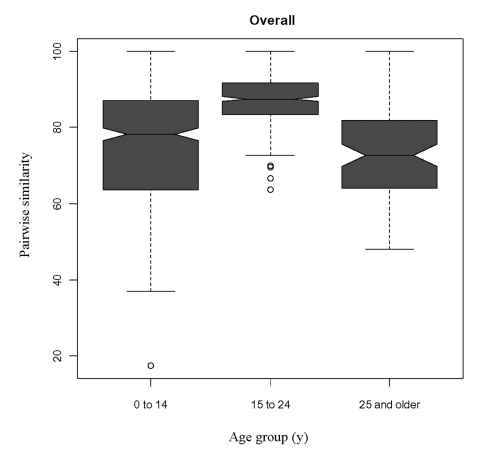
Box-plot of mean pairwise similarities demonstrating the genetic relatedness of serogroup C strains for persons <14 years (32 strains), persons 15–24 years (28 strains), and adults ∃>25 years (14 strains) during 1992–1999. The lower, central, and upper horizontal lines in the box indicate the 25th, 50th, and 75th percentiles. The outliers, as defined as the 25th or 75th quartile ±1.5x the interquartile range, are plotted as circles. Notches of box plots that do not overlap indicate a statistically significant difference at the <0.05 level.

In the pairwise similarity analysis, serogroup C infection in those 15–24 years of age was caused by isolates that were more highly genetically related than were serogroup C isolates from other age groups (Table 3 and Figure 2). For example, the median (25th and 75th percentiles) pair-wise similarity during 1992 through 1999 for serogroup C isolates that caused infections in those 15 to 24 years of age was 87.5% (83.3%; 91.7%), in contrast to 78.3% (63.6%, 87.0%) and 72.7% (64.0%, 81.8%) for persons <15 and >25 years of age, respectively (p < 0.01 for the comparisons of those 15–24 years of age versus each of the other two age groups).

**Table 3:** Pairwise similarities (% similarity) of serogroup C strains, by age group and time period

Period	Age group (y)	No. isolates	25%	50% (median)	75%	Mean	SD	p value^a^
1992–1999	<14	32	63.6	78.3	87.0	75.4	14.9	<0.01
	15–24	28	83.3	87.5	91.7	87.6	7.4	
	>25	14	64.0	72.7	81.8	74.2	12.1	
1992–1997	<14	26	60.9	76.2	84.6	73.1	15.3	<0.01
	15–24	20	81.8	88.0	90.9	86.2	7.2	
	>25	10	61.5	72.7	83.3	73.2	13.7	
1998–1999	<14	6	82.6	87.0	95.7	88.9	6.9	<0.01
	15–24	8	98.9	100	100	97.0	5.4	
	>25	4	70.7	72.7	78.2	76.1	8.1	

^a^Kruskal-Wallis rank sum test for the comparison of those 15–24 years of age versus each of the two other age groups.

**Figure 3:**
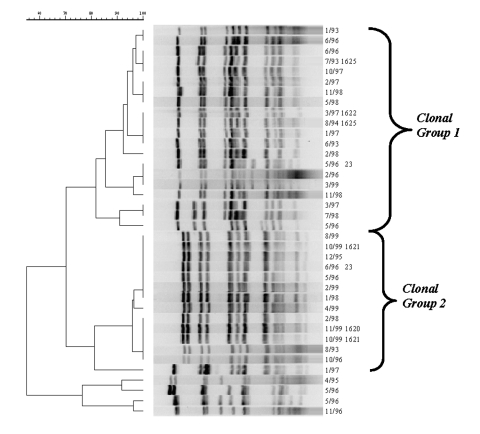
Pulsed-field gel electrophoresis patterns of meningococcal serogroup Y strains isolated from persons >25 years during 1992–1999. Culture date and sequence type are listed to the right of the dendrogram.

Ninety-two percent (70/76) of the serogroup Y isolates were classified into one of two clonal groups (Figure 3). The cophenetic correlation for the serogroup Y dendrogram was 92.3%. The proportion of clonal group 2 serogroup Y strains increased from 7.7% (1/13) in 1992 to 1993; 20.0% (2/10) in 1994 to 1995; 17.9% (5/28) in 1996 to 1997; to 52.0% (13/25) in 1998-99 (p <0.01, exact test for trend). Among adults ∃>25 years of age, clonal group 2 strains increased from 25.0% (1/4) of the case-patients in 1992 to 1993; 33.3% (1/3) in 1994 to 1995; 23.5% (4/17) in 1996 to 1997; and 57.1% (8/14) in 1998 to 1999 (p = 0.28).

Among the 14 serogroup Y isolates on which MLST was performed (Tables 1, 2), clonal group 1 strains belonged to ST 23, ST 1622, or ST 1625, and clonal group 2 belonged to ST 23, ST 1620, and ST 1621. Overall, the serogroup Y clonal group 1 and 2 strains tested belonged to the ST-23 complex. The nonclonal group strains had unrelated STs.

## Discussion

From 1992 to 1997, the increasing incidence of invasive meningococcal disease among persons aged 15–24 years of age was caused primarily by a clonal group of serogroup C strains that belonged to the ST-11 complex. These strains were much more genetically related than the serogroup C isolates, which were causing infection in the two other age groups. In general, ST-11 complex strains belong to the ET-37 complex (*7*), which has been associated with outbreaks and epidemics (*8*,*14*). Although the meningococcal incidence decreased in this age group in the late 1990s, a unique PFGE-defined serogroup C clone that had not been previously detected emerged in 1999. Among patients infected with serogroup Y isolates, a shift from one ST-23 Y clonal group to another occurred over time.

The risk of meningococcal infection depends on a variety of strain, host, and environmental factors (*15*). During epidemics, which are generally clonal, the proportion of cases that occur in adolescents and young adults often rises, in addition to an increased incidence in other age groups (*3*). This pattern is believed to be caused, at least in part, by the introduction of a new strain to which the population has little immunity. The increase in adolescents and young adults is similar to the pattern we observed in Maryland, although no epidemic was associated with it. Analogous to the epidemic setting, we hypothesized that the increase in those 15–24 years of age was due to the introduction of a serogroup C clone to which this group had not previously been exposed. We expected the increase to be caused in large part by this new clone, as adolescents and young adults typically otherwise have a low risk for meningococcal infection. This is in contrast to younger children and older adults, who in general are more susceptible to meningococcal infection and therefore might be expected to be infected with a broader range of strains, in addition to the newly introduced clone (*2*,*16*).

Although our study had not begun early enough to determine whether clonal group 1 serogroup C strains were truly recently introduced into Maryland, the observation that serogroup C infection in those 15 to 24 years of age was caused by more highly related strains generally supports our hypothesis. The emergence of a new PFGE-defined clone in adolescents in 1999 was somewhat surprising since the meningococcal incidence was decreasing in this age group during that time. The shift from one ST-23 complex serogroup Y clonal group to another during the 1990s may also have been due to the changes in population immunity that occur over time because of the circulation of *N. meningitidis* in the community. However, since we did not study whether the two clonal groups differed in cell surface immunogens, this hypothesis remains speculative.

The molecular epidemiology of meningococcal infection has historically been determined by MEE (*3*,*15*). Recently, however, MLST has been validated as an alternative method to MEE. We found that PFGE further discriminated among strains with the same ET. For example, MLST could not discriminate between the two serogroup C PFGE-defined clones in the 1997 and 1999 outbreaks. This finding was similar to those of a recent study in which meningococcal isolates with the same ET were found to have distinct PFGE patterns (*8*). Taken together, these data indicate that PFGE and MLST are complementary methods for studies of the molecular epidemiology of *N. meningitidis* infection (*8*,*13*).

In summary, epidemiologic trends in invasive meningococcal disease in Maryland from 1992 to 1999 were associated with specific PFGE-based genotypes of serogroup C and Y strains. Additional studies are needed to determine whether future changes in meningococcal incidence are associated with variations in the strains causing invasive disease.
